# Extended Genetic Diversity of Bovine Viral Diarrhea Virus and Frequency of Genotypes and Subtypes in Cattle in Italy between 1995 and 2013

**DOI:** 10.1155/2014/147145

**Published:** 2014-06-22

**Authors:** Camilla Luzzago, Stefania Lauzi, Erika Ebranati, Monica Giammarioli, Ana Moreno, Vincenza Cannella, Loretta Masoero, Elena Canelli, Annalisa Guercio, Claudio Caruso, Massimo Ciccozzi, Gian Mario De Mia, Pier Luigi Acutis, Gianguglielmo Zehender, Simone Peletto

**Affiliations:** ^1^Department of Veterinary Science and Public Health, University of Milan, Via Celoria 10, 20133 Milan, Italy; ^2^Department of Clinical Sciences “Luigi Sacco”, Section of Infectious Diseases, University of Milan, Via G.B. Grassi 74, 20157 Milan, Italy; ^3^Istituto Zooprofilattico Sperimentale dell'Umbria e delle Marche, Via G. Salvemini 1, 06126 Perugia, Italy; ^4^Istituto Zooprofilattico Sperimentale della Lombardia e dell'Emilia Romagna, Via Bianchi 9, 25124 Brescia, Italy; ^5^Istituto Zooprofilattico Sperimentale della Sicilia “A. Mirri”, Via G.Marinuzzi 3, 90129 Palermo, Italy; ^6^Istituto Zooprofilattico Sperimentale Piemonte, Liguria e Valle D'Aosta, Via Bologna 148, 10154 Torino, Italy; ^7^Department of Infectious Parasitic and Immunomediated Diseases, National Institute of Health, Viale Regina Elena 299, 00161 Rome, Italy; ^8^University of Biomedical Campus, Via Álvaro del Portillo, 200, 00128 Rome, Italy

## Abstract

Genetic typing of bovine viral diarrhea virus (BVDV) has distinguished BVDV-1 and BVDV-2 species and an emerging putative third species (HoBi-like virus), recently detected in southern Italy, signaling the occurrence of natural infection in Europe. Recognizing the need to update the data on BVDV genetic variability in Italy for mounting local and European alerts, a wide collection of 5′ UTR sequences (*n* = 371) was selected to identify the frequency of genotypes and subtypes at the herd level. BVDV-1 had the highest frequency, followed by sporadic BVDV-2. No novel HoBi-like viruses were identified. Four distribution patterns of BVDV-1 subtypes were observed: highly prevalent subtypes with a wide temporal-spatial distribution (1b and 1e), low prevalent subtypes with a widespread geographic distribution (1a, 1d, 1g, 1h, and 1k) or a restricted geographic distribution (1f), and sporadic subtypes detected only in single herds (1c, 1j, and 1l). BVDV-1c, k, and l are reported for the first time in Italy. A unique genetic variant was detected in the majority of herds, but cocirculation of genetic variants was also observed. Northern Italy ranked first for BVDV introduction, prevalence, and dispersion. Nevertheless, the presence of sporadic variants in other restricted areas suggests the risk of different routes of BVDV introduction.

## 1. Introduction

Bovine viral diarrhea virus (BVDV), a widespread pathogen of cattle, was first described in North America in 1946 [1, 2]. Seroprevalence rates between 36% and 88% have subsequently been reported since the 1960s in North America, Europe, Australia, and East Africa. The endemic diffusion of BVDV persists in geographic areas where no systematic control measures have been implemented and it reflects the pathogenic mechanisms of BVDV through which the virus can establish both transient and persistent infections. Persistent infected (PI) animals, originating from a transient infection of pregnant cows or born from PI cows, shed large amounts of virus throughout their lives and ensure viral persistence in the host population. Besides the previous maintenance strategy, a peculiar biological characteristic of BVDV contributes to iatrogenic diffusion of infection. In fact, two different biotypes coexist and are differentiated by their effect on cell culture in cytopathic (cp) and noncytopathic biotypes (ncp). Contamination of fetal bovine serum (FBS) by the ncp biotype has long been known and remains a recognized risk factor for the worldwide distribution of BVDV [3, 4]. Because FBS is used in the production of vaccines and other biological products, the global trade of infected FBS products is a potential source of transboundary spread of BVDV.

BVDV belongs to the* Pestivirus *genus of the Flaviviridae family. Genetic typing of BVDV isolates distinguishes two recognized species: BVDV-1 and BVDV-2. To date, 17 potential BVDV-1 subtypes have been identified [5–10] and BVDV-2 strains can be clustered into at least three subtypes [5, 11, 12]: BVDV-1a to BVDV-1q and BVDV-2a to BVDV-2c, respectively. A putative third bovine species, referred to as HoBi-like virus or BVDV-3 [13], comprises viral strains recently detected in FBS batches originating mainly from South America, as well as from Australia, Canada, Mexico, and the United States [14]. Unlike the widespread diffusion of HoBi-like viruses via FBS, natural infection in cattle has been reported in Southeast Asia [15], in South America [16, 17], and in two dairy herds in southern Italy [18, 19]. The Italian cases represent the first identification of HoBi-like virus in cattle in Europe.

In addition to the risk of transboundary spread of BVDV through potentially infected FBS, the genetic diversity of the virus in a given geographic area has been largely influenced by animal movement within countries and/or introduction from other countries, as recently observed in Switzerland [27], Italy [21], and England and Wales [28], showing that the introduction and spatial distribution of BVDV can also be influenced by livestock management practices.

Nucleotide sequencing is a rapid and inexpensive diagnostic tool for unambiguous typing of all bovine pestiviruses. Broad systematic surveillance of BVDV genetic variability is advisable for updating data on the distribution and frequency of emerging genotypes and subtypes in BVDV endemic countries. The aim of this study was therefore to analyze a representative and epidemiologically well-characterized collection of BVDV sequences from Italian cattle. The previous genotyping studies were carried out on a limited number of isolates [20, 22–24, 29, 30]. The present study represents a comprehensive collection of Italian isolates obtained from all cattle breeding areas over a time period spanning nearly two decades (1995–2013). Genetic variability was determined to identify the frequency of genotypes and subtypes with resolution at the herd level.

## 2. Materials and Methods 

### 2.1. Samples and Data Set

The material comprised samples sent to laboratories between 1995 and 2013 for routine testing because of suspected BVDV infection and for screening in voluntary herd control programs. The BVDV positive samples and the derived sequences were selected on the basis of the following criteria: (1) sequences obtained from different animals, (2) known herd and locality where the strain was isolated and sampling dates, and (3) sequences representative of all Italian BVDV subtypes and genotypes. A total of 371 BVDV 5′UTR sequences were included, 272 of which were novel sequences and 99 were BVDV Italian sequences retrieved from published peer-reviewed journals. Samples were obtained from 357 cattle and 14 bulk milk specimens collected from 259 Italian cattle herds from around the country. Samples were obtained from 164 dairy herds, 40 beef herds, and 11 mixed production systems, and 44 were undetermined. Only one sequence from each herd was available for the majority of the herds (*n* = 210); 2 to 20 sequences were included for the 49 remaining herds. The localities of origin of the strains were grouped into four macroareas: the North, the Center, the South, and the Islands (Sicily and Sardinia). In detail, the North macroarea comprised the regions of Emilia Romagna (*n* = 41), Lombardy (*n* = 108), Piedmont (*n* = 130), Valle d'Aosta (*n* = 1), and Veneto (*n* = 18); the Center, Latium (*n* = 6), Marches (*n* = 4), Tuscany (*n* = 2), and Umbria (*n* = 15); the South, Basilicata (*n* = 4), Calabria (*n* = 5), Campania (*n* = 4), and Puglia (*n* = 2); the Islands, Sicily (*n* = 29) and Sardinia (*n* = 2).

### 2.2. RT-PCR and Sequencing

Viral RNA of the 272 novel sequences was extracted from original biological samples (*n* = 261) and growth medium after passage in cell culture (*n* = 11). Reverse transcription and PCR assays targeting a 288 bp region of 5′UTR of BVDV were performed using previously described primers by Letellier et al. [31], with the exception of strains collected in Sicily, which were tested using primers by Vilcek et al. [32]. The samples collected in the Center and the South macroareas were also tested by primers for atypical* Pestivirus* [14]. For the BVDV-1 subtypes identified for the first time in Italy, a 428 bp region encoding autoprotease N^pro^ was amplified using nested PCR, as previously described [20, 29].

For each sample, the amplicons of the expected sizes were purified and sequenced using forward and reverse primers by cycle sequencing using a Big Dye Terminator version 1.1 Cycle Sequencing kit (Applied Biosystems, CA, USA) and an ABI PRISM 3130 sequencing device or sent for outsource sequencing (Primm).

### 2.3. Phylogenetic Analysis

All the sequences were aligned with BVDV reference strains retrieved from GenBank representative of BVDV-1, BVDV-2, and HoBi-like virus using Clustal X; manual editing was performed with Bioedit software version 7.0 (freely available at http://www.mbio.ncsu.edu/bioedit/bioedit.html). Phylogeny was estimated by the neighbor-joining algorithm (NJ) and the maximum likelihood (ML) method.

The evolutionary model that best fitted the data (GTR + I + G) was selected using an information criterion implemented in JmodelTest [33] (freely available at http://darwin.uvigo.es/software/jmodeltest.html).

NJ analysis of the 5′UTR was performed using molecular evolutionary genetics analysis (MEGA version 5) [34], with 1000 bootstrap replicates; ML tree was reconstructed with Phyml version 3.0 (http://www.atgc-montpellier.fr/phyml) with 1000 bootstrap replicates.

The sequences within the same genotypes and subtypes from the same herd and from different herds were compared, and the percentage of nucleotide similarity of pairwise evolutionary distances was calculated using MEGA version 5.

## 3. Results

**Figure 1:**
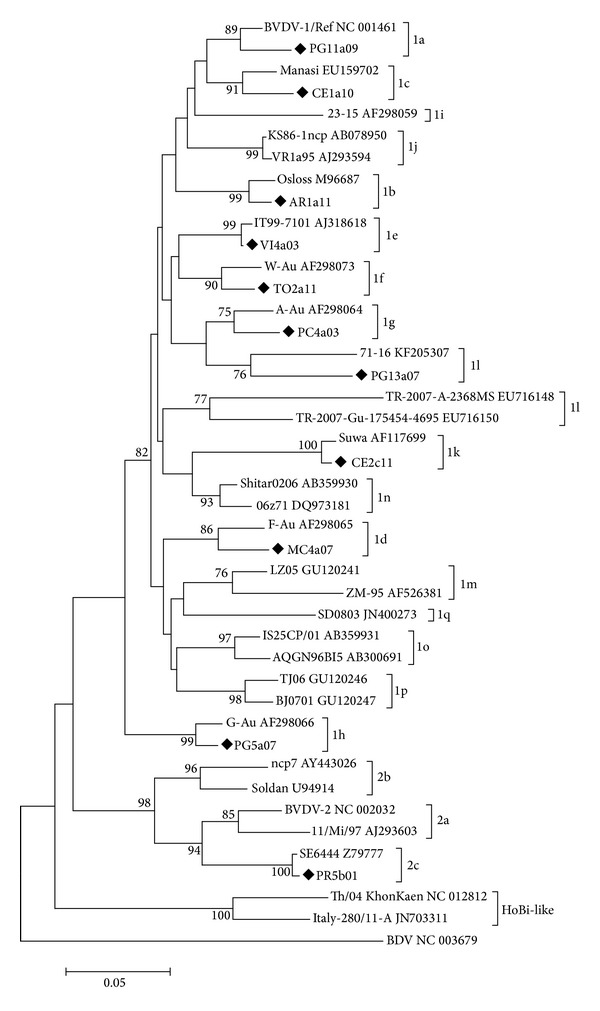
Phylogenetic tree based on the 5′-UTR of selected Italian sequences representative of all BVDV genotypes and subtypes detected between 1995 and 2013 and reference BVDV-1, BVDV-2, and HoBi-like strains. Molecular evolutionary genetics analyses were performed with MEGA5 using the NJ method. Distances were computed using the Kimura 2-parameter model. Bootstrap values > 70% are shown. Published sequences and references are identified by GenBank accession number (available at http://www.ncbi.nlm.nih.gov/pubmed/). The symbol “◆” indicates selected novel nucleotide sequences of BVDV Italian strains.

A total of 269 out of 272 sequences obtained in the present study were typed as BVDV-1 upon analysis of 5′UTR to reference strains and three were classified as BVDV-2, using both NJ and ML methods. No HoBi-like viruses were identified in the Center and South macroareas using primers described by Xia et al. [14] and Letellier et al. [31], in northern Italy and Sardinia using primers by Letellier et al. [31], and in Sicily using primers described by Vilcek et al. [32]. A selection of Italian BVDV sequences representative of all BVDV genotypes and subtypes detected were reported in the NJ phylogenetic tree of the 5′UTR region, together with reference strains (Figure 1). A phylogenetic tree of all the sequences was also represented (see Supplementary Figure 1 available online at http://dx.doi.org/10.1155/2014/147145).

With regard to both the novel and the published sequences, 357 (96.2%) sequences belonged to BVDV-1, ten (2.7%) to BVDV-2, and four (1.1%) to HoBi-like virus. The BVDV-1 sequences belonged to 11 different subtypes (a, b, c, d, e, f, g, h, j, k, and l). The frequency of genotypes and subtypes is summarized in Table 1.

Among the subtypes identified, BVDV-1c, k, and l are reported here for the first time in Italy; typing of these subtypes was confirmed by N^pro^ region analysis (data not shown). Phylogenetic analysis showed that the BVDV-2 strains clustered with the subtypes a and c according to [12].

BVDV-1b and BVDV-1e showed the highest frequency at both the animal and the herd levels, being detected in 43.9% and 27.5% of herds, respectively. The frequency of BVDV-1a, d, f, g, h, and k was less than 10%; BVDV-1c, j, and l were sporadically obtained from single herds. The frequency of BVDV-2 was low at both the animal and the herd levels (2.7% at the herd level, Table 1).

**Figure 2:**
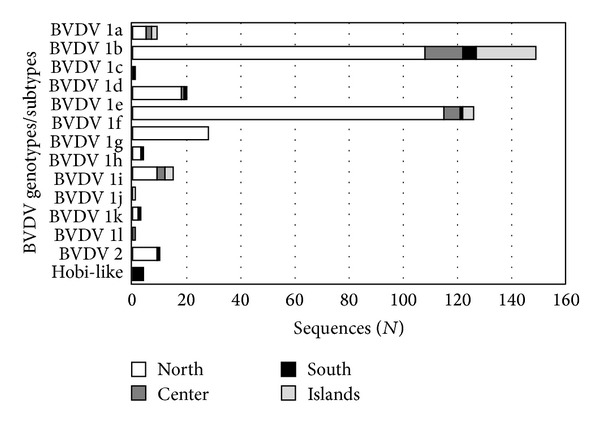
Frequency of BVDV genotypes and subtypes in the four Italian macroareas.

The North macroarea, where cattle population density is highest, accounted for 298 (80.3%) BVDV sequences, the Center for 27 (7.3%), the South for 15 (4%), and the Islands (Sicily and Sardinia) for 31 (8.4%). When differentiated by geographic distribution, BVDV-1 and BVDV-2 were present in the North, BVDV-1 in the Center and the Islands, and BVDV-1, BVDV-2, and HoBi-like virus in the South. Nine different BVDV-1 subtypes were detected in the North, six in the Center and the South, and four in the Islands. The more frequent subtypes (BVDV-1b and 1e) were distributed across the entire country, whereas the less frequent subtypes (BVDV-1a, d, g, h, and k) were present in two or three macroareas, except for BVDV-1f, which was limited to the North (Table 1 and Figure 2). The most frequent subtypes were also distributed across all the years, whereas the lower prevalence subtypes and genotypes were detected sporadically (Supplementary Table 1).

The percentage of sequence similarity of pairwise evolutionary distances within BVDV genotypes and subtypes ranged from 83.1% to 100%. Forty of the 49 herds with more than one sequence had the same genotype or subtype (37 BVDV-1, two BVDV-2, and one HoBi-like). In addition, 100% sequence identity of BVDV-1 within herds was observed in 19/37 herds within a range of sampling between 1 day and 14 months. In 18/37 herds, the nucleotide percentage similarity was ≤ 99.4% within a range of sampling between 2 months and 12 years (Table 2). The remaining nine herds had different genotypes or subtypes, without showing any relationship between the number of sequences analyzed and the time of sampling (Table 3).

## 4. Discussion 

Molecular typing studies have demonstrated a wide genetic diversity of BVDV in Italy [20, 22–24, 29, 30], where its geographic distribution is influenced by animal movement within the country and/or importation from other countries [21]. Besides the two known BVDV species, a third species, referred to as HoBi-like or BVDV-3, has recently been detected in two herds in Italy [18, 19]. Surveillance of Italian BVDV isolates is therefore advisable to update data on the national genetic variability for mounting local and European-wide alerts.

A comprehensive collection of new BVDV sequences from all cattle breeding areas in Italy was investigated by phylogenetic analysis and compared against a selection of reference sequences of known genotypes and subtypes retrieved from public genetic databases. A total of 371 sequences have been included over a time span of 18 years (1995–2013) and were analyzed using the neighbor-joining and the maximum likelihood methods. At the genotype level, BVDV-1 had the highest frequency, followed by sporadic BVDV-2 in a limited area of the North (Lombardy and Emilia Romagna) and only once in the South. In the present study, the most recent detection of BVDV-2 dates back to 2004, encompassing nearly a decade during which the potential risk of transmission by BVDV-2 contaminated biological products had been reported [35, 36]. No novel HoBi-like virus was identified using panpestivirus primers described by Letellier et al. [31] that are able to detect HoBi-like virus [37, 38]; moreover, in central and southern Italy, a protocol for atypical* Pestivirus* detection [14] has been also applied. Regarding Sicily, we cannot exclude failure of HoBi-like viruses detection especially in samples with a low viral load, as previously reported for the primer pair used [4, 37]. The sporadic frequency of the HoBi-like virus in Italy was also confirmed by the National Reference Center for* Pestivirus* that did not detect any other HoBi-like strains on testing an additional collection of 450 BVDV field samples (Giammarioli personal communication, 2014).

The genetic diversity of BVDV-1 in Italy is increasing, as compared to previously published findings [20, 30]. Here we report the circulation of three additional subtypes, namely, BVDV-1c, 1k, and 1l, the last classified as the French 1l subtype described by [6], accounting for 11 out of the 17 BVDV-1 subtypes recognized worldwide [9, 10]. The increased phylogenetic diversity of BVDV-1 and the presence of new viral subtypes during the years were also reported in other European countries, especially due to the analysis of broader collection of BVDV isolates and/or introduction and movement of cattle from Europe [6, 28]. Different BVDV-1 subtypes are predominant in European countries; concerning the Italian neighboring countries, the most prevalent subtypes are BVDV-1e in France, BVDV-1e and 1h in Switzerland, BVDV-1h in Austria, and BVDV-1d and 1f in Slovenia [6, 27, 39, 40]. Interestingly, BVDV-1e is predominant in Piedmont, the Italian region close to the French border that is also characterized by the major commercial introduction of cattle from France.

Four frequency and distribution patterns of BVDV-1 subtypes were identified in Italy: high prevalent subtypes with a wide temporal-spatial distribution (BVDV-1b and 1e), low prevalent subtypes with a widespread geographic distribution (BVDV-1a, 1d, 1g, 1h, and 1k), low prevalent subtypes in restricted geographic areas (BVDV-1f in the North), and sporadic subtypes detected only in single herds (BVDV-1c, 1j, and 1l). The North macroarea showed the highest genetic variability, with nine out of 11 BVDV-1 subtypes and the cocirculation of BVDV-2, confirming the predominant role of this area in BVDV introduction into Italy from other European countries [21]. Nevertheless, the identification of some sporadic genetic variants restricted to the Center (BVDV-1l) or the South (BVDV-1c and HoBi-like) and the presence of eight BVDV genotypes and subtypes in the South, despite the low frequency of total sequences, suggest that BVDV has been likely introduced in Italy also through different commercial livestock flows or the use of contaminated biological products.

The genetic variability among BVDV isolates of the same subtype in the same herd was investigated in all the herds where more than one sequence was available. Two major genetic patterns were observed: the presence of herd-specific strains, also for prolonged periods (up to 14 months), and the presence of genetic variability of the same BVDV subtype within single herds, particularly within several months after the first date of sampling, likely indicating a new infection with a different strain. In this respect, further molecular analysis and investigation of epidemiological links among farms are needed to assess and gain insight into the frequency of reinfection and/or the molecular evolution of BVDV strains detected in the same herd and between herds, as recently applied by [41]. Moreover, a third less frequently observed genetic pattern was the presence of different subtypes or genotypes within the same herd at the same date of sampling, indicating BVDV cocirculation, possibly through exposure to multiple viral sources. Cocirculation of different BVDV subtypes was detected in both milk and beef production systems, confirming that the diversity of viral strains in the Italian cattle population influences the variability also at the herd level.

## 5. Conclusion

This comprehensive overview of the genetic variability of BVDV strains circulating in Italy highlights a marked genetic diversity. The temporal-spatial distribution of BVDV variants suggests the risk of different routes of BVDV introduction and dispersion, through different commercial livestock flows or the use of contaminated biological products, likely related to the lack of coordinated control measures. Also, it highlights the importance of phylogenetic studies for genetic characterization and for reconstruction of the evolutionary relationships between strains through which the ecological and epidemiological mechanisms driving such genetic heterogeneity may be elucidated. Advanced phylogenetic analysis of the evolutionary dynamics of BVDV strains present in a population can aid in tracing transmission chains and prevents and controls infections and sources of reinfections.

## Supplementary Material

Table S1: Temporal distribution of BVDV genotypes and subtypes in Italy between 1995 and 20013.
Figure S1: Phylogenetic tree based on the 5'-UTR of 371 Italian sequences representative of all BVDV genotypes and subtypes detected between 1995 and 2013.

## Figures and Tables

**Table 1:** Frequency of BVDV genotypes and subtypes in Italy.

Genotype/subtype	Total sequences number (%)	Herd∗ number (%)	Years	Geographic origin∗∗	Published/total sequences (*n*)	References
BVDV-1a	9 (2.4)	9 (3.4)	2000–2009	NCI	3/9	[20]
BVDV-1b	149 (40.2)	115 (43.9)	1995–2013	NCSI	52/149	[20–24]
BVDV-1c	1 (0.3)	1 (0.4)	2010	S	0/1	Not published
BVDV-1d	20 (5.4)	16 (6.1)	1995–2010	NCS	5/20	[22, 23]
BVDV-1e	126 (34)	72 (27.5)	1996–2013	NCSI	22/126	[20–24]
BVDV-1f	28 (7.5)	17 (6.5)	1999–2012	N	1/28	[23]
BVDV-1g	4 (1.1)	4 (1.5)	2002–2010	NS	1/4	[20]
BVDV-1h	15 (4.0)	14 (5.3)	1996–2011	NCI	3/15	[21–23]
BVDV-1j	1 (0.3)	1 (0.4)	1995	N	1/1	[22]
BVDV-1k	3 (0.8)	3 (1.1)	2001–2011	NS	0/3	Not published
BVDV-1l	1 (0.3)	1 (0.4)	2007	C	0/1	Not published
BVDV-2	10 (2.7)	7 (2.7)	1995–2004	NS	7/10	[20, 22]
HoBi-like	4 (1.1)	2 (0.8)	2007–2011	S	4/4	[18, 19, 25, 26]

*Herds with different BVDV genotypes or subtypes are counted for each virus type.

**N: northern Italy, C: central Italy, S: southern Italy, and I: Islands.

**Table 2:** Sequence similarity (%) of pairwise distances within herds with ≥2 animals/sequences belonging to the same BVDV-1 subtypes.

Sequence similarity (%)	BVDV-1 subtype	Herd identification	Sequences in each herd (*n*)	Temporal range of collection (months)	Herd production system∗
100	1b	RG7	2	2	nd
100	1b	BG23	2	12^a^	D
100	1b	PD3	2	12^a^	nd
100	1b	CO2	2	1^b^	D
100	1b	CN29	2	1^b^	nd
100	1b	AT3	2	1^b^	B
100	1b	CN8	2	1	B
100	1b	TO3	2	1^b^	D
100	1b	TO17	3	13	M
100	1d	CN20	2	1^b^	B
100	1e	RG2	2	8	nd
100	1e	CR22	2	1^b^	D
100	1e	AL2	2	1^b^	M
100	1e	CN24	2	6	D
100	1e	TO8	5	1^b^	D
100	1e	TO14	8	12	B
100	1e	TO19	7	1^b^	D
100	1f	CN2	3	14	M
100	1h	NO1	2	1	D
99.4–100	1e	VI7	4	12^a^	nd
99.4	1b	RG6	2	1^b^	nd
99.4	1b	TO23	2	3	D
99.4	1d	CN19	2	1^b^	D
99.4	1f	AT2	2	1^b^	B
98.8–100	1b	LO2	4	3	D
98.8–100	1f	CN1	7	14	D
98.8	1f	CN5	2	2	D
98.2–100	1b	RG4	4	2	nd
98.2	1b	RM1	2	12^a^	D
98.2	1b	TR1	2	12^a^	B
97.1	1b	CR13	2	12^a^	D
96.5	1b	RM2	2	12^a^	D
94.7–100	1b	LC1	5	129	D
93.6–100	1e	CN16	3	10	B
93–100	1b	RG3	3	3	nd
90.1	1e	CN17	2	13	D
88.9–100	1e	TO12	7	19	M

*D: dairy herd, B: beef herd, M: mixed farm, and nd: not determined.

^a^Data available only for year of sample collection.

^b^Samples collected on the same day.

**Table 3:** Herds with ≥2 animals/sequences belonging to different genotypes and subtypes.

Genotype	Herd identification	Total sequences number	Temporal range of collection (months)	Herd production system∗
BVDV-1b, 1e	TO9	2	1∗∗	D
BVDV-1b, 1e	CN7	20	1	B
BVDV-1b, 1f	CN6	2	3	B
BVDV-1b, 1k	CS2	3	12	D
BVDV-1b, 2	BS33	2	1∗∗	D
BVDV-1d, 1e	NO2	3	21	M
BVDV-1d, 1e	MN4	2	1∗∗	D
BVDV-1e, 1f	CN3	5	1∗∗	D
BVDV-1e, 1h	LC2	2	19	D

*D: dairy herd, B: beef herd, and M: mixed farm.

**Samples collected on the same day.
