# Supplementary material for: Extended Genetic Diversity of Bovine Viral Diarrhea Virus and Frequency of Genotypes and Subtypes in Cattle in Italy between 1995 and 2013
Source: Biomed Res Int. 2014 Jun 22;2014:147145. doi: 10.1155/2014/147145 (PMC4090534; doi:10.1155/2014/147145)

Supplementary Table 1. Temporal distribution of BVDV genotypes and subtypes in Italy.

| Year | BVDV genotype and subtype* | | | | | | | | | | | | |
| --- | --- | --- | --- | --- | --- | --- | --- | --- | --- | --- | --- | --- | --- |
|  | 1a | 1b | 1c | 1d | 1e | 1f | 1g | 1h | 1j | 1k | 1l | 2 | HoBi-like |
| 1995 |  |  |  |  |  |  |  |  |  |  |  |  |  |
| 1996 |  |  |  |  |  |  |  |  |  |  |  |  |  |
| 1997 |  |  |  |  |  |  |  |  |  |  |  |  |  |
| 1998 |  |  |  |  |  |  |  |  |  |  |  |  |  |
| 1999 |  |  |  |  |  |  |  |  |  |  |  |  |  |
| 2000 |  |  |  |  |  |  |  |  |  |  |  |  |  |
| 2001 |  |  |  |  |  |  |  |  |  |  |  |  |  |
| 2002 |  |  |  |  |  |  |  |  |  |  |  |  |  |
| 2003 |  |  |  |  |  |  |  |  |  |  |  |  |  |
| 2004 |  |  |  |  |  |  |  |  |  |  |  |  |  |
| 2005 |  |  |  |  |  |  |  |  |  |  |  |  |  |
| 2006 |  |  |  |  |  |  |  |  |  |  |  |  |  |
| 2007 |  |  |  |  |  |  |  |  |  |  |  |  |  |
| 2008 |  |  |  |  |  |  |  |  |  |  |  |  |  |
| 2009 |  |  |  |  |  |  |  |  |  |  |  |  |  |
| 2010 |  |  |  |  |  |  |  |  |  |  |  |  |  |
| 2011 |  |  |  |  |  |  |  |  |  |  |  |  |  |
| 2012 |  |  |  |  |  |  |  |  |  |  |  |  |  |
| 2013 |  |  |  |  |  |  |  |  |  |  |  |  |  |

* colored boxes indicate detection of the BVDV genotype and subtype.

Supplementary Figure 1. Phylogenetic tree based on the 5’-UTR of 371 Italian sequences representative of all BVDV genotypes and subtypes detected between 1995 and 2013 and reference BVDV-1, BVDV-2, and HoBi-like strains. Molecular evolutionary genetics analyses were performed with MEGA5 using the neighbor-joining method. The genotypes and subtypes detected in Italy have been highlighted.


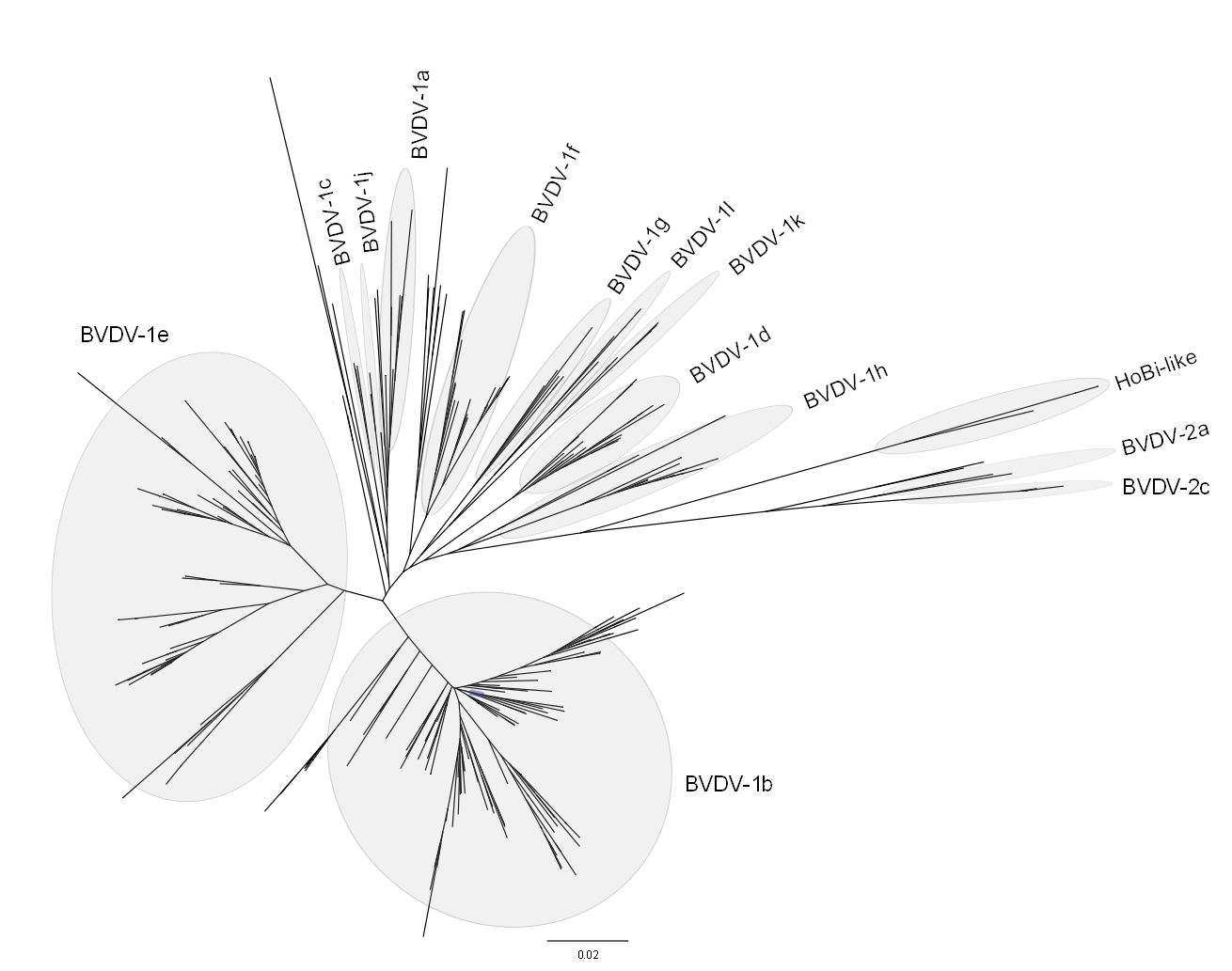

Supplement: Supplementary file 1 — Table S1: Temporal distribution of BVDV genotypes and subtypes in Italy between 1995 and 20013. Figure S1: Phylogenetic tree based on the 5'-UTR of 371 Italian sequences representative of all BVDV genotypes and subtypes detected between 1995 and 2013. [file 147145.f1.docx]
